# Exposing the Masquerade of Nocardia otitidiscaviarum Pneumonia: A Case Report

**DOI:** 10.7759/cureus.67849

**Published:** 2024-08-26

**Authors:** Sandhya Nallamotu, Mahith S Reddy

**Affiliations:** 1 Department of Medicine, Kasturba Medical College, Manipal, Manipal, IND

**Keywords:** multidrug therapy, infection microbiology, bacterial infectious disease, pneumonia, culture sensitivity, opportunistic pathogen, immunocompetent, nocardia otitidiscaviarum, pulmonary nocardiosis

## Abstract

We present a rare case of an immunocompetent 49-year-old male agriculturalist from India diagnosed with *Nocardia otitidiscaviarum* pneumonia. *Nocardia* species are ubiquitous gram-positive, partially acid-fast bacilli that predominantly infect immunocompromised individuals. Only 0.3% to 2.9% of all nocardiosis cases are attributed to *N. otitidiscaviarum*. The patient presented with a 25-day history of wet cough and high-grade fever, with bilateral bronchial breath sounds on chest auscultation and findings consistent with pneumonia on chest X-ray. During hospitalization, multiple treatment revisions were made. On admission, empiric antibiotic therapy against community-acquired pneumonia was initiated. Later, GeneXpert sputum testing for *Mycobacterium tuberculosis* complex (MTBC) was positive for MTBC DNA. Suspected tuberculosis with a secondary infection prompted a treatment switch to antitubercular therapy (ATT) along with meropenem. Despite changes to treatment, the patient continued to deteriorate with no signs of clinical improvement. ATT with meropenem was discontinued when a repeat GeneXpert for MTBC was negative, ruling out tuberculosis. Slow-growing bronchial wash culture identified the rare pathogen* N. otitidiscaviarum*, prompting an urgent referral to a specialized Infectious Diseases team. Treatment was then tailored according to antibiotic resistance-sensitivity testing. Targeted multidrug antibiotic therapy with trimethoprim-sulfamethoxazole and amikacin against *N. otitidiscaviarum* facilitated gradual clinical improvement. This case underscores the importance of considering uncommon pathogens in differential diagnosis and highlights the critical role of microbiological diagnostics in guiding effective treatment. Drug resistance and changing bacterial pathogenicity trends must not be overlooked. The round-about antibiotic treatment changes in this case point to the necessity for faster diagnostic methods in identifying *Nocardia* species. Further research into rapid diagnostic methods and up-to-date treatment guidelines are warranted to optimize outcomes in nocardiosis management.

## Introduction

Nocardiosis is an infection caused by bacteria of the genus *Nocardia*, belonging to the family Nocardiaceae. *Nocardia species* are gram-positive, weakly acid-fast, aerobic filamentous bacilli found ubiquitously in organic-rich soil and standing water [1,2]. Human nocardiosis infection manifests as cutaneous, pulmonary, central nervous system, or disseminated infections [3]. No cases of human-to-human or animal-to-human transmission have been reported so far. Environmental exposure to *Nocardia species* in contaminated soil and water by inhalation or direct inoculation transmits the pathogen [1]. Pulmonary nocardiosis constitutes 60-70% of nocardiosis cases and predominantly affects immunocompromised individuals [3]. The incidence of nocardiosis appears to be increasing, although data remains limited [4].

Recent molecular methods, such as the sequencing of the 16S rRNA gene, have enhanced the accurate identification of *Nocardia* species, which is crucial for guiding appropriate antibiotic therapy [2]. Primary pathogens causing nocardiosis include* Nocardia asteroides*, *Nocardia farcinica*, and *Nocardia brasiliensis*, whereas *Nocardia otitidiscaviarum* is less frequently implicated [3-9]. Variability in antimicrobial susceptibility and limited data on rare *Nocardia* species create a substantial knowledge gap for physicians managing these infections. Current understanding relies heavily on laboratory in-vitro studies. Furthermore, widespread antibacterial resistance mechanisms among *Nocardia species* complicate the choice of empiric antimicrobial treatments [10]. Here, we present a rare case of *N. otitidiscaviarum* pneumonia.

## Case presentation

**Figure 1:**
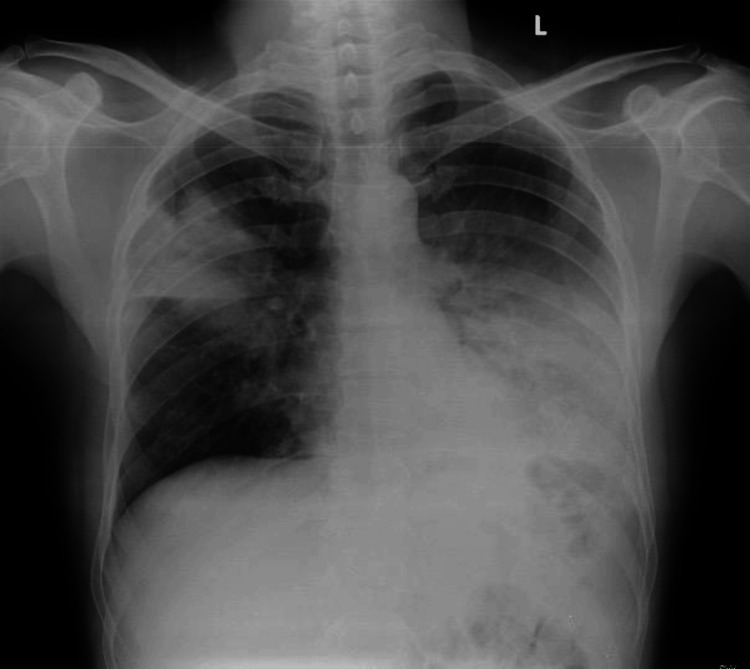
Chest X-ray on admission shows right upper lobe and left lower lobe consolidation.

A 49-year-old previously healthy male agriculturalist presented with a 25-day history of cough and fever. Initial treatment at a local hospital did not alleviate symptoms, prompting his referral to Kasturba Hospital, Manipal. On admission, the patient presented with persistent high-grade fever with chills, rigors, and a productive cough with white sputum. Apart from a high-grade fever, all other vital signs were within normal expected limits. Respiratory examination revealed bronchial breath sounds over the left infra-scapular and infra-axillary lung areas and the right mammary and axillary lung areas. Other systemic examinations were within normal expected limits. Lab investigations for anti-human immunodeficiency virus, anti-hepatitis C virus, and hepatitis B HbsAg were all non-reactive, confirming that the patient had no indications of immunodeficiency-related illnesses and thus was most likely immunocompetent. The patient’s renal and liver function lab tests were within normal limits. Chest X-ray showed right upper lobe and left lower lobe consolidation suggestive of pneumonia (Figure 1).

Empiric antibiotics for community-acquired pneumonia with piperacillin, tazobactam, and azithromycin were initiated. Later, based on a positive sputum sample on GeneXpert testing for *Mycobacterium tuberculosis *complex (MTBC), antitubercular treatment (ATT) for suspected tuberculosis replaced the empiric antibiotics. Despite one week of hospital admission and ATT, the patient’s fever persisted without much discernable clinical or lab study improvement (Table 1).

**Table 1:** Lab trends from hospital admission to discharge. TLC: total leukocyte count; DLC: differential leukocyte count; CRP: C-reactive protein; ESR: erythrocyte sedimentation rate

Lab Test	Day 1	1 week	6 weeks	7 weeks
TLC (cells/μL)	12,500	16,800	4,100	4,300
DLC				
Neutrophil	73%	85%	63.7%	41%
Lymphocyte	10.7%	4.5%	23.2%	29.7%
Monocyte	15%	9.3%	10.2%	27.6%
Eosinophil	0.6%	0.5%	2.7%	0.9%
Basophil	0.7%	0.7%	0.2%	0.8%
Platelet count (cells/μL)	634,000	83,000	165,000	205,000
Procalcitonin (ng/L)	0.5883%	-	-	-
CRP (mg/L)	146.04	-	31.36	-
ESR (mm/hour)	80	61	70	-

Meropenem was subsequently added to treatment, on suspicion of a secondary bacterial infection. Sputum stain microscopy showed minimal bacteria consistent with normal oropharyngeal flora. Sputum culture reports found no *M. tuberculosis*, *Aspergillosis*, or fungal growth.

Given the negative detection of the pathogen on simple sputum sample testing, a bronchoalveolar lavage for deeper respiratory bronchial wash samples was ordered. Bronchoalveolar lavage was done to ensure adequate infective bacterial counts and lower levels of normal oropharyngeal flora were obtained within bronchial wash samples to allow for possible aerobic culture growth of the causative organism. Bronchial wash aerobic culture growth confirmed *N. otitidiscaviarum*. Antibiotic susceptibility testing (AST) results are shown in Table 2.

**Table 2:** Nocardia otitidiscaviarum culture antibiotic susceptibility testing result.

Antibiotic susceptibility testing	Sensitive or resistant
Ampicillin/Amoxicillin	Resistant
Cefotaxime/Ceftriaxone	Resistant
Amikacin	Sensitive
Amoxicillin-clavulanic acid	Sensitive
Ciprofloxacin/Ofloxacin	Sensitive
Trimethoprim-sulfamethoxazole	Sensitive
Erythromycin	Sensitive
Gentamycin	Sensitive
Tetracycline/Doxycycline	Sensitive
Imipenem	Sensitive
Linezolid	Sensitive

The patient’s bronchial wash sample was also sent for a second GeneXpert MTBC test, which helped conclusively rule out MTBC. A false-positive GeneXpert MTBC is difficult to verify and resolve because it is a molecular assay that identifies both live and non-viable MTBC DNA. However, our microbiologists felt the first GeneXpert to be a false positive because subsequent aerobic culture growth was identified as *Nocardia*, and a repeat GeneXpert done was negative. Further speculation of MTBC was put to rest by the lack of clinical improvement in the patient despite three weeks of ATT. Thus, ATT and meropenem were discontinued.

The patient still showed no signs of symptomatic improvement. No other complications such as cavitation, abscess formation, pleural effusion, or empyema were identified on Chest X-ray, and no new findings were elicited on the respiratory clinical examination. Therefore, it was speculated that ineffective treatment was the primary cause of the patient’s lack of expected improvement. Treatment was revised based on AST results shown in Table 2. An urgent referral to the Infectious Disease Department was sent for an expert opinion on further medical therapy.

**Figure 2:**
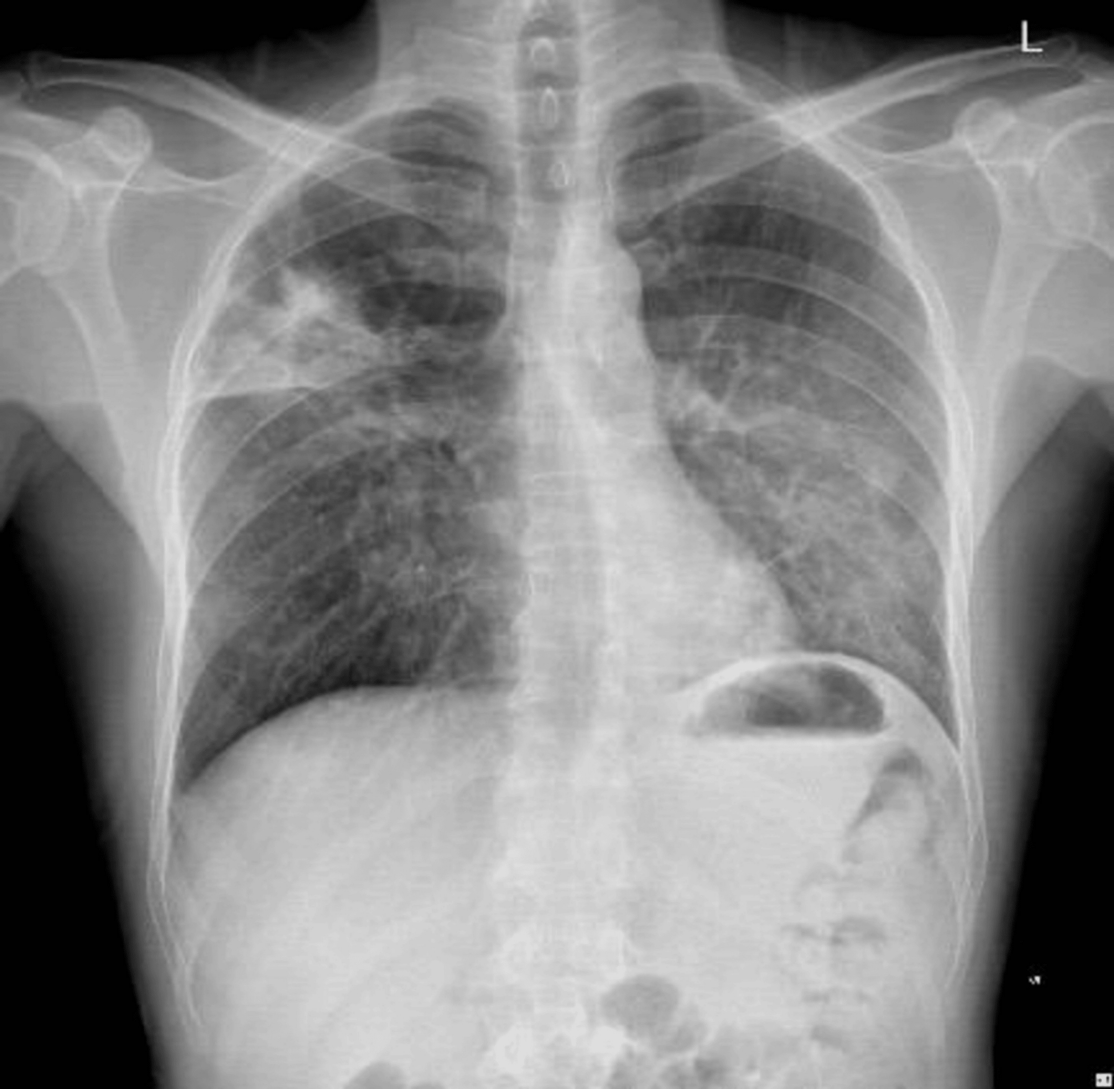
Chest X-ray on discharge shows improvement of Nocardia otitidiscaviarum pneumonia.

After review by Infectious Disease specialists, the patient was promptly started on a 14-day regimen of trimethoprim-sulfamethoxazole (TMP-SMX) and amikacin, resulting in clinical and symptomatic improvement (Figure 2, Table 1).

He was discharged on oral TMP-SMX for six months, aligning with recommendations for immunocompetent patients.

## Discussion

*N. otitidiscaviarum*, previously known as *Nocardia caviae*, is estimated to account for only 0.3% to 2.9% of all reported nocardiosis cases. A lower prevalence in the environment compared with other *Nocardia *species may contribute to the lower incidence of *N. otitidiscaviarum* infections [11,12]. Although considered less pathogenic to humans than other *Nocardia species*, it is important to diagnose and treat cases of *N. otitidiscaviarum* infections in a timely manner. Based on factors such as different strain variability, inoculum size, and infection route, the pathogenicity of* N. otitidiscaviarum* is never constant and must be duly assessed in every individual case. Without any pathognomonic signs, symptoms, and imaging features, it can be difficult to clinically diagnose *N. otitidiscaviarum* infections [13].

Smear and culture of sputum, wound drainages, or bronchial washes are the main methods of diagnosis [13]. It can take up to two to three weeks to identify *N. otitidiscaviarum* by culture due to its slow growth [14]. Polymerase chain reaction, 16S rDNA sequencing, and mass spectroscopy are more rapid identification methods for* N. otitidiscaviarum*.

In a characteristic case of an opportunistic nocardiosis infection,* N. otitidiscaviarum* pneumonia was reported by Srivastava et al. in an immunocompromised patient who was treated with monotherapy of TMP-SMX. Much like the case we presented, this patient also underwent two to three antibiotic treatment revisions until *Nocardia *grew on aerobic culture. *Nocardia *species identification was done by matrix-assisted laser desorption ionization-time of flight mass spectrometry. Despite having started and taken TMP-SMX monotherapy for 2.5 months, unfortunately, this patient succumbed to the infection after a long three-to-four-month struggle. Key points to note of similarity are that physicians treating this patient faced the same time-to-diagnosis setback in being able to start specific management within the current recommendations which led to multiple antibiotic therapy revisions. Something our medical team considers learning from this example is for future physicians treating nocardial infections to consider multidrug therapy rather than a monotherapy approach. Patients do not always respond to conventional treatment, therefore, metagenomic next-generation sequencing studies can facilitate faster decision-making by providing crucial data about *Nocardia species *resistance patterns. The immune status of the patient and the interventions taken are factors that play a role in the high mortality rates associated with nocardiosis. As Srivastava et al. note in their case report, we concur that though the reported prevalence in India is around 1.47% to 1.9%, the reported epidemiology of nocardiosis seems to be actively evolving. This case is yet another example that brings to light the importance of early diagnosis in nocardiosis, especially for immunocompromised patients [15].

Once diagnosed, treating *N. otitidiscaviarum* infection requires a focused long course of drug therapy. The suggested duration of antibiotic therapy is six months in immunocompetent patients and up to one year in immunosuppressed individuals [16]. *N. otitidiscaviarum* is generally known to have good susceptibility to TMP-SMX. Many isolates also show susceptibility to amikacin and fluoroquinolones and were resistant to beta-lactams such as ampicillin, amoxicillin-clavulanic acid, and imipenem [13]. In-vitro studies have also proven that *N. otitidiscaviarum* is responsive and susceptible to linezolid, but data from in-vivo studies are lacking. The incidence of hematological toxicity risk increases within four weeks of linezolid use posing another drawback to its use in the treatment of *N. otitidiscaviarum* [17]. Despite recent evidence, suggesting that the species have developed increasing resistance and demonstrated inconsistent susceptibility to TMP-SMX, sulfonamides remain the antibiotic treatment of choice [6,9,11,15,18]. Previously successful treatment regimens may have become obsolete given new drug resistance developments. Therefore, it is necessary to properly test for the patient’s bacterial culture sensitivity reports before starting a treatment regimen. Although there is no recommended optimal treatment protocol to be followed for *N. otitidiscaviarum*, a combination of sulphonamides and amikacin with a carbapenem or a third-generation cephalosporin can be suggested for severe or disseminated infections [1,11,18-20].

Pulmonary nocardiosis, as presented in this case report, holds a mortality rate of 15% to 30%, and up to 50% mortality in severe cases [17,19]. In light of these harrowing figures, it is crucial for the international community of medical professionals to create an optimal regimen for the rapid diagnosis and treatment of nocardiosis.

## Conclusions

Nocardial infections present formidable challenges in clinical management. Current diagnosis relies on slow-growing cultures, delaying effective treatment and often leading to frequent changes in antibiotic therapy. Antibiotic changes increase the risk of new antibiotic resistance. This case highlights these hurdles in the diagnosis and management of *N. otitidiscaviarum* pneumonia in an immunocompetent, otherwise healthy, middle-aged man. Thus, we advise physicians to consult with specialists such as an infectious disease team, as was done in this case, when faced with nocardial infections. Though rarely implicated in non-opportunistic human infection, the variable pathogenicity of *N. otitidiscaviarum* emphasizes clinical awareness of the evolving microbiological landscape. Therefore, we stress the need for rapid diagnostic tools and standardized treatment protocols to improve diagnostic time, optimize therapy, and prevent antibiotic resistance in the management of nocardiosis.
